# The Hidden Costs of a Bloodless Field: A Systematic Review of the Complications of Tourniquet Use in Total Knee Arthroplasty

**DOI:** 10.7759/cureus.95885

**Published:** 2025-11-01

**Authors:** Chan Khin, Olive Kyaw

**Affiliations:** 1 Trauma and Orthopaedics, St George's University Hospitals NHS Foundation Trust, London, GBR; 2 Trauma and Orthopaedics, University Hospitals Sussex NHS Foundation Trust, Brighton, GBR

**Keywords:** limb occlusion pressure, pain, quadriceps dysfunction, randomised controlled trial, total knee arthroplasty, tourniquet, venous thromboembolism, wound complications

## Abstract

Pneumatic tourniquets are widely used in total knee arthroplasty (TKA) to improve visualization and cement fixation, yet their potential harms remain debated. This study systematically evaluated complications associated with tourniquet use in primary TKA, focusing on wound complications as the primary outcome and venous thromboembolism (VTE), pain, quadriceps dysfunction, transfusion, and systemic adverse events as secondary outcomes. MEDLINE (Medical Literature Analysis and Retrieval System Online), Embase, and Cochrane Controlled Register of Trials (CENTRAL) were searched from January 2000 to July 2025. Eligible randomized controlled trials (RCTs) compared (i) tourniquet vs no tourniquet, (ii) full-duration vs limited/timed use, or (iii) alternative pressure strategies. Two reviewers independently screened, extracted data, and assessed risk of bias (RoB 2). Random-effects meta-analyses were performed where appropriate. Certainty of evidence was appraised using GRADE (Grading of Recommendations, Assessment, Development, and Evaluations). Twenty-six reports (25 unique RCT datasets; 3,183 patients, 2001-2024) were included. Across 1,940 patients in 15 RCT comparisons, tourniquet use showed a non-significant trend toward more wound complications (risk ratio (RR) 1.31, 95%CI 0.73-2.36; moderate certainty, I²=20.9%). VTE across 2,144 patients in 15 RCTs showed no significant difference (RR 1.09, 95%CI 0.72-1.64; low certainty). Transfusion in three RCTs (199 patients) showed no clear difference (RR 0.45, 95%CI 0.19-1.04; low certainty). Six RCTs (~500 patients) consistently reported greater early pain and quadriceps dysfunction with tourniquet. Rare but serious adverse events (e.g., compartment syndrome, vascular injury) were documented. In primary TKA, routine full-duration tourniquet use does not reduce VTE or transfusion requirements, is associated with early pain and quadriceps dysfunction, and may increase wound morbidity. Pressure-optimised or selective approaches may mitigate harms while preserving technical benefits. Routine use should be reconsidered in favour of tailored strategies.

## Introduction and background

Total knee arthroplasty (TKA) is among the most frequently performed and successful orthopaedic procedures worldwide for end-stage degenerative knee disease [1,2]. Despite its proven efficacy, perioperative concerns such as blood loss, wound complications, venous thromboembolism (VTE), and postoperative pain continue to shape surgical practice. The pneumatic tourniquet has been widely adopted for more than four decades to create a bloodless field, improving intraoperative visualisation, facilitating cement penetration, and reducing apparent blood loss [3,4].

Nevertheless, routine tourniquet use remains controversial. Randomized controlled trials (RCTs) have shown that tourniquet inflation may reduce visible intraoperative bleeding but does not consistently decrease total blood loss and may instead contribute to wound morbidity, delayed rehabilitation, quadriceps dysfunction, and thromboembolic events [5-8]. Selective strategies, such as cementation-only inflation, have been proposed to balance safety with technical benefits, but findings are inconsistent and include higher transfusion rates in some trials [9-11]. More recently, attention has turned to optimising cuff pressure, with trials evaluating limb occlusion pressure (LOP)-based protocols versus fixed thresholds [12-14].

The rationale for focusing on complications is that outcomes such as wound morbidity, functional recovery, pain, and thromboembolic risk directly affect patient recovery and quality of life, whereas intraoperative blood loss alone is less patient-centred. These outcomes are particularly relevant in the context of modern enhanced recovery protocols.

Although several systematic reviews and meta-analyses have examined the role of tourniquet use in TKA, most have focused on intraoperative blood loss, transfusion requirements, and early functional outcomes, often pooling data from both randomised and observational studies [15,16]. RCTs provide more reliable evidence on safety and recovery, yet relatively few reviews have systematically synthesised the broader spectrum of tourniquet-related complications.

A recent large-scale meta-analysis by Boutros et al. (2025) included 50 RCTs and provided valuable insights into perioperative blood loss, pain, and early functional recovery [17]. However, their review gave less emphasis to detailed safety outcomes, such as wound complications, VTE, quadriceps dysfunction, and other adverse events. These complications are of particular concern, as they directly affect patient safety, rehabilitation, and long-term outcomes.

The present review, therefore, synthesises evidence exclusively from RCTs with a primary focus on complications of tourniquet use, including wound complications, VTE, pain, quadriceps dysfunction, transfusion, and other adverse events. By concentrating on safety endpoints rather than blood loss alone, this review complements recent large-scale analyses and provides a complication-focused synthesis to guide clinical practice.

## Review

Methods

Eligibility Criteria

We included RCTs enrolling adults undergoing primary TKA that compared pneumatic tourniquet use with either no tourniquet, limited or timed tourniquet application (for example, cementation-only), or alternative pressure strategies (for example, limb occlusion pressure-based protocols versus fixed thresholds). Trials were required to report at least one complication outcome as a primary or secondary endpoint. We excluded non-randomised studies, revision or unicompartmental knee arthroplasty, and conference abstracts without full text.

Information Sources and Search Strategy

We searched MEDLINE (Medical Literature Analysis and Retrieval System Online), Embase, and Cochrane Controlled Register of Trials (CENTRAL) (all via Ovid) for records published between January 1, 2000, and July 31, 2025. Reference lists of included studies and relevant reviews were screened to identify additional trials. The Ovid search combined controlled vocabulary and free-text terms related to TKA, tourniquet use, and complications; the complete strategy is provided in the Appendices. 

Protocol and Registration

The review was registered prospectively with the International Prospective Register of Systematic Reviews (PROSPERO) (CRD420251148456) on September 14, 2025. Any amendments were limited to clarifying subgroup definitions (tourniquet duration and cuff pressure) and sensitivity analyses for rare events and were finalized prior to data synthesis.

Selection Process

Two reviewers (OK and CK) independently screened titles/abstracts and subsequently assessed full texts for eligibility. Disagreements were resolved by consensus. The Preferred Reporting Items for Systematic Reviews and Meta-Analyses (PRISMA) 2020 guidelines were followed for study selection.

Data Collection and Data Items

Two reviewers (OK and CK) independently extracted data using a piloted form. We recorded study characteristics, participant demographics, intervention details, and outcomes. Complications were classified into five domains: (1) VTE (deep vein thrombosis, pulmonary embolism), (2) wound complications (infection, blistering, dehiscence, delayed healing, wound drainage), (3) local effects related to tourniquet use (thigh pain, nerve palsy, compartment syndrome), (4) systemic complications (cardiovascular, renal, respiratory), and (5) functional consequences (quadriceps dysfunction, reoperation, manipulation under anaesthesia).

Risk of Bias Assessment

Risk of bias was evaluated with the Cochrane RoB 2 tool [18] across five domains (randomization process; deviations from intended interventions; missing outcome data; outcome measurement; and selection of reported results). Assessments were performed independently by two reviewers, with consensus adjudication.

Statistical Analysis

For dichotomous outcomes, risk ratios (RRs) with 95% confidence intervals (CIs) were calculated. A continuity correction of 0.5 was applied when a single trial arm had zero events; double-zero studies were excluded from meta-analysis and summarized narratively. Pooled estimates were obtained using random-effects models (restricted maximum likelihood (REML); DerSimonian-Laird for sensitivity). Peto odds ratios (PORs) were additionally calculated for rare events. Heterogeneity was assessed with the χ² test, I² statistic, and τ², with I² >50% considered substantial. Subgroup analyses were pre-specified by tourniquet strategy (continuous vs limited/cement-only), cuff pressure regimen (fixed vs limb occlusion pressure-based), and timing of inflation (selective vs throughout). Sensitivity analyses included exclusion of high-risk-of-bias trials and leave-one-out re-analyses. Small-study effects were to be evaluated with funnel plots and Egger’s regression if ≥10 studies contributed. Certainty of evidence was judged using the GRADE (Grading of Recommendations, Assessment, Development, and Evaluations) approach.

Certainty of Evidence

Certainty of evidence for each outcome was appraised using the GRADE approach [19]. We applied GRADE to rate certainty per outcome (high, moderate, low, very low), with footnotes specifying reasons for downgrading.

Results

Study Selection

**Figure 1:**
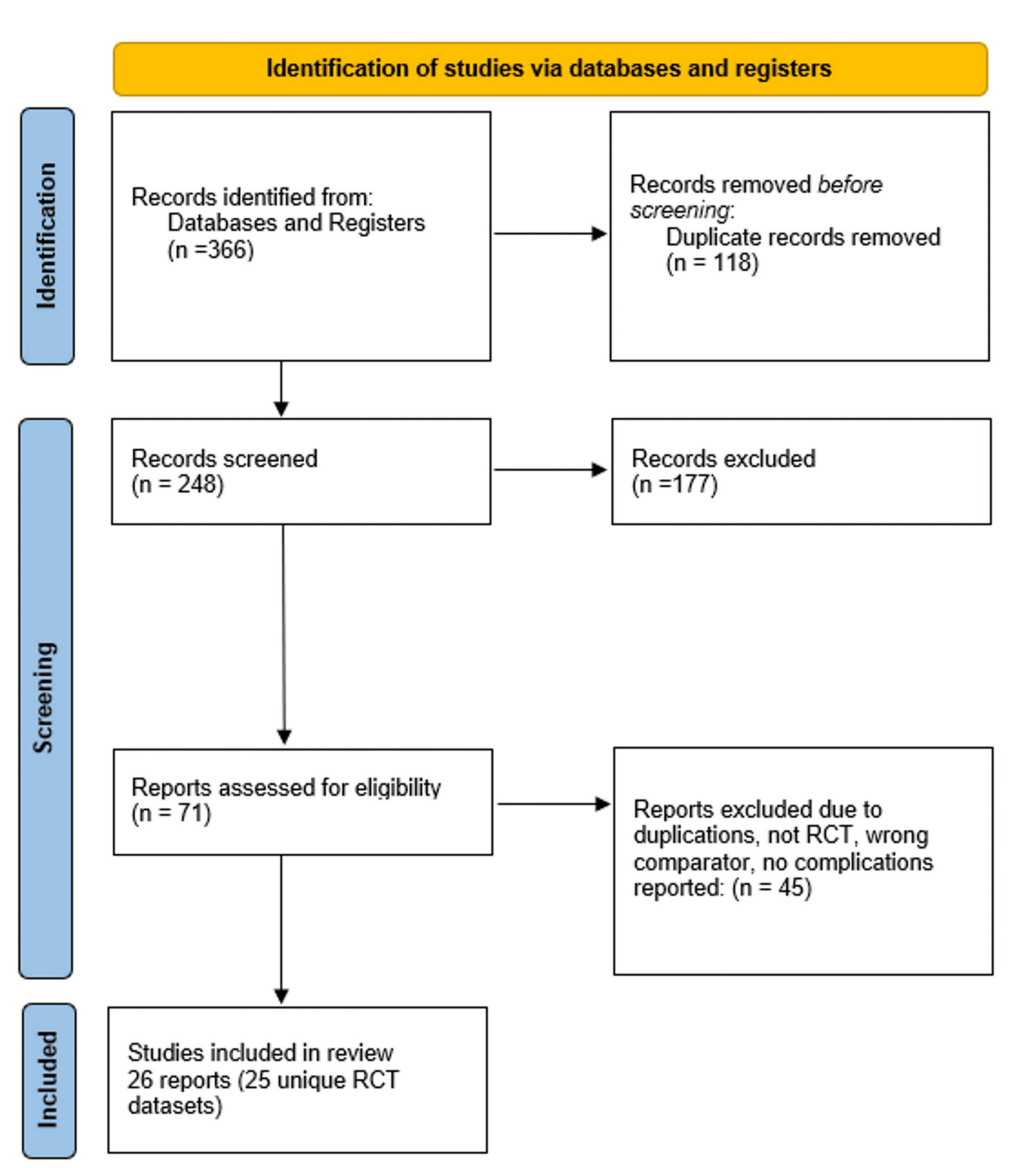
PRISMA 2020 flow diagram of study selection Flow of information through the systematic review process. A total of 366 records were identified through database searching. After removal of duplicates, 71 records were screened by title and abstract. Full texts were assessed for eligibility, with 45 excluded for reasons such as wrong study design, non-relevant comparator, or absence of complication outcomes. In total, 26 randomised controlled trials were included in the final review. One report (Rantasalo et al., 2021 [20]) is a prespecified secondary/subset analysis of Palanne et al., 2021 [21] and was not counted as an independent dataset in pooled analyses.

The search yielded 366 records. After de-duplication, 248 records were screened by title/abstract, and 71 full texts were assessed. A total of 26 reports (representing 25 unique RCT datasets) met the inclusion criteria (Figure 1).

Study Characteristics

Trials were published during 2001-2024 across Asia, Europe, North America, and Australia. All included studies were prospective RCTs (Table 1). Most compared full-duration vs no tourniquet, with others comparing cementation-only or different pressure strategies. Sample sizes ranged from 28 to 395. Mean ages were 60s-70s, BMI was 24-32 kg/m², and mostly American Society of Anesthesiologists (ASA) I-II.

**Table 1:** Characteristics of included randomised controlled trials (N = 25*) Some effect estimates are not shown at the study level due to absent reporting; pooled estimates are provided in meta-analysis figures. Dennis et al., 2016 [7] and Liu et al., 2017 [32] used contralateral limb designs (outcomes per limb). *One report (Rantasalo et al., 2021 [20]) is a prespecified secondary/subset analysis of Palanne et al., 2021 [21] and was not counted as an independent dataset in pooled analyses. AOP: arterial occlusion pressure; ASA: American society of anaesthesiologist; BMI: body mass index; LOP: limb occlusion pressure; NR: not reported in the source publication, SBP: systolic blood pressure; TQ: tourniquet; TXA: tranexamic acid.

Study (Author, Year)	Country	Sample size (N)	Study arms	Intervention details	Mean age (years)	Sex (M/F)	BMI (kg/m²)	ASA I–II / ≥III
Chaiyakit et al., 2024 [11]	Thailand	90	Tourniquet throughout vs Cementation-only	Conventional tourniquet vs limited cementation	68.3 vs 67.0	5/40 vs 7/38	26.8 vs 26.6	19/25/1 vs 17/27/1
Diri et al., 2023 [22]	Syria	62	Tourniquet throughout vs Cementation-only	Full duration vs cementation-only	67.4 vs 65.5	9/22 vs 12/19	28.1 vs 28.9	NR
Tan et al., 2023 [8]	China	50	Tourniquet vs No tourniquet	Full duration vs no cuff	70 vs 72	7/18 vs 6/19	25.5 vs 26.0	NR
Dong et al., 2024 [23]	China	130	Tourniquet vs No tourniquet	Full duration vs none	69.2 vs 67.5	16/49 vs 12/53	27.6 vs 26.7	NR
Dragosloveanu et al., 2023 [24]	Romania	190	Tourniquet vs No tourniquet	Full duration vs none	68.4 vs 67.5	49/51 vs 49/45	NR	NR
Ran et al., 2022 [25]	China	184	Tourniquet vs No tourniquet	Full duration vs none	70.9 vs 70.9	23/69 vs 20/72	25.4 vs 25.2	40/52 vs 42/50
Stocks et al., 2023 [26]	United States	97	Tourniquet vs No tourniquet	Tourniquet vs none	64.4 vs 65.0	34/35 vs 15/33	34.9 vs 34.1	NR
Hung et al., 2023 [27]	Taiwan	110	Tourniquet vs No tourniquet	Standard use vs none	73.1 vs 71.4	22/33 vs 22/33	24.7 vs 23.9	36/29 vs 38/17
Palanne et al., 2021 [21]	Finland	395	Tourniquet vs No tourniquet	Standard use vs none	63.5 vs 64.0	66/134 vs 78/117	30.6 vs 30.2	139 vs 141 (ASA I–II); 61 vs 54 (ASA ≥III)
Yasin et al., 2020 [28]	India	103	Tourniquet vs No tourniquet	Full duration vs none	Majority 61–70 yrs	25/26 vs 27/25	NR	NR
Kim et al., 2019 [29]	Korea	160	Lower vs Conventional pressure	SBP+120 vs SBP+150 mmHg	NR	NR	NR	NR
Tarwala et al., 2014 [10]	United States	79	Tourniquet vs Cementation-only	Full duration vs cementation-only	66.1 vs 64.6	13/22 vs 14/22	29.9 vs 31.4	NR
Vandenbussche et al., 2002 [30]	France	80	Tourniquet vs No tourniquet	Tourniquet vs none	72.5 vs 68.5	9/31 vs 16/24	NR	NR
Goel et al., 2019 [6]	India	200	Tourniquet vs No tourniquet	Full duration vs none	66.0 vs 65.5	50/50 vs 48/52	30.9 vs 31.3	NR
Dennis et al., 2016 [7]	USA	28 pts (56 limbs)	Tourniquet vs No tourniquet (contralateral limb)	Tourniquet limb vs contralateral limb	62 both	16/12 vs 16/12	NR	NR
Tai et al., 2012 [5]	Taiwan	72	Tourniquet vs No tourniquet	Full duration vs none	72.1 vs 71.5	9/27 vs 8/28	28.6 vs 27.9	NR
Mittal et al., 2012 [9]	Australia	65	Long vs short duration	Skin incision → cement vs cement-only	66.6 vs 67.5	9/25 vs 6/25	32.6 vs 32.5	NR
Huang et al., 2017 [31]	China	150	3 arms	TQ + TXA vs No TQ + TXA vs TQ only	66.2 / 65.1 / 65.8	18/32, 16/34, 15/35	25.1 / 24.4 / 24.7	13/25/12 vs 11/33/6 vs 16/28/6
Liu et al., 2017 [32]	China	52 pts (104 knees)	Tourniquet vs No tourniquet (contralateral limb)	Tourniquet limb vs contralateral	NR	16M/36F each	NR	NR
Olivecrona et al., 2012 [12]	Sweden	164	LOP vs Standard pressure	LOP-based vs fixed >225 mmHg	NR	NR	NR	NR
Zhang et al., 2010 [33]	China	60	Tourniquet vs No tourniquet	Full duration vs none	72 vs 71	8/22 vs 11/19	25 vs 26	NR
Tetro and Rudan, 2001 [34]	Canada	63	Tourniquet vs No tourniquet	Tourniquet vs none	69.8 both	15/18 vs 11/19	NR	NR
Natesan et al., 2024 [14]	India	311	LOP vs SBP+150 mmHg	Optimised pressure vs conventional	62.3 vs 63.4	22/132 vs 35/122	30.5 vs 29.1	NR
Wu et al., 2022 [13]	China	138	3 arms	AOP vs SBP+100 vs 300 mmHg	71.7 / 70.3 / 71.7	19/31, 18/32, 19/31	24.0 / 23.9 / 23.6	26/18/6 vs 33/16/1 vs 28/19/3
Yi et al., 2021 [4]	China	150	3 arms	Full vs No TQ vs Cement-only	68.4 / 68.0 / 68.7	7/43, 8/42, 7/43	26.1 / 25.3 / 25.9	35/15, 38/12, 36/14

Study Design

Most trials compared tourniquet use throughout the procedure versus no tourniquet. A second group compared conventional full-duration application against limited or timed use (e.g., cementation-only inflation or early release). Several more recent trials investigated alternative pressure strategies (e.g., limb occlusion pressure, systolic blood pressure +120/150 mmHg, or fixed 300 mmHg).

Tourniquet Versus No Tourniquet RCTs

A total of 15 RCTs compared standard tourniquet application throughout the procedure with no tourniquet use (n ≈ 1,862 patients) (Table 2).

**Table 2:** Randomised controlled trials – tourniquet vs no tourniquet Rantasalo et al., 2021 [20] is a subset analysis of Palanne et al., 2021 [21]. Dennis et al., 2016 [7] and Liu et al., 2017 [32] used contralateral limb designs (outcomes per limb). AE: adverse events; CI: confidence interval; DVT: deep vein thrombosis; GI: gastrointestinal; KSS: Knee Society Score; MI: myocardial infarct; MRI: magnetic resonance imaging; MUA: manipulation under anaesthesia; NR: not reported in the source publication; OR: odds ratio; PE: pulmonary embolism; POD: postoperative day; ROM: range of motion; TQ: tourniquet; VTE: venous thromboembolism

Study (Author, Year)	Total participants	Arm A	Arm B	Key Findings / Complications Reported
Tan et al., 2023 [8]	50	Tourniquet (n=25)	No tourniquet (n=25)	VTE: 2/25 vs 1/25; Wound complications: 6/25 vs 0/25; Infection: 1/25 vs 0/25; UTI: 0/25 vs 1/25
Dong et al., 2024 [23]	130	Tourniquet (n=65)	No tourniquet (n=65)	VTE: 14/65 vs 14/65; Other wound/skin/cerebral events: 7/65 vs 2/65
Dragosloveanu et al., 2023 [24]	190	Tourniquet (n=96)	No tourniquet (n=94)	VTE: 5/96 vs 2/94; Arthrofibrosis: 2/96 vs 3/94; Wound complication (readmission): 1/96 vs 0/94
Ran et al., 2022 [25]	184	Tourniquet (n=92)	No tourniquet (n=92)	Knee swelling: 23/92 vs 5/92; Hypoproteinaemia: 41/92 vs 26/92; Severe pain: 33/92 vs 12/92; Opioid use (72 h): 64/92 vs 48/92
Stocks et al., 2023 [26]	97	Tourniquet (n=49)	No tourniquet (n=48)	MRI-confirmed quadriceps strain: 22/49 vs 13/48 (OR 2.7, 95% CI 1.1–6.7, p=0.028)
Hung et al., 2023 [27]	110	Tourniquet (n=55)	No tourniquet (n=55)	VTE: 0/55 vs 1/55; Quadriceps recovery slower in tourniquet group
Rantasaloet al., 2021 [20]	391	Tourniquet (n=197)	No tourniquet (n=194)	Pain AE: 36/197 vs 23/194; Swelling: 10/197 vs 12/194; Superficial infection: 0/197 vs 3/194; PE: 0/197 vs 1/194; DVT: 1/197 vs 0/194; Muscle vein thrombosis: 1/197 vs 0/194; Nerve damage: 1/197 vs 0/194; MI: 0/197 vs 1/194; Arrhythmia: 0/197 vs 1/194; Cardiac insufficiency: 1/197 vs 0/194; MUA <90° ROM: 23/197 vs 15/194
Yasin et al., 2020 [28]	103	Tourniquet (n=51)	No tourniquet (n=52)	Mild tourniquet pain/blistering in TQ group; No transfusion required in either arm
Zhang et al., 2010 [33]	60	Tourniquet (n=30)	No tourniquet (n=30)	No complications reported
Tai et al., 2012 [5]	72	Tourniquet (n=36)	No tourniquet (n=36)	Transfusion: 2/36 vs 2/36; Thigh pain (higher in TQ, POD4 p=0.014); Knee pain (higher in TQ, POD4 p=0.033); No major AE (DVT/nerve/infection)
Dennis et al., 2016 [7]	28 pts / 56 limbs (contralateral limb design; outcomes per limb)	Tourniquet limb (n=28)	Contralateral no-TQ limb (n=28)	One PE (no-TQ group), 1 asymptomatic DVT (no-TQ limb), 1 wound dehiscence (TQ limb, required surgery)
Liu et al., 2017 [32]	104	Tourniquet limb (n=52)	Contralateral no-TQ limb (n=52)	Pain ↑ with TQ; swelling ↑ with TQ; wound ooze: 63.5% vs 26.9%; erythema: 55.8% vs 28.8%; blisters: 17.3% vs 3.8%; ecchymosis: 28.8% vs 9.6%; 1 deep infection (TQ); DVT: 4 vs 4; ROM & KSS similar
Vandenbussche et al., 2002 [30]	80	Tourniquet (n=40)	No tourniquet (n=40)	↑ blood loss without TQ; less pain at 6h and better flexion early without TQ; no major complications
Goel et al., 2019 [6]	200	Tourniquet (n=100)	No tourniquet (n=100)	Thigh pain/numbness: 13/100 vs 17/99; DVT: 0/100 vs 1/99; Wound complication: 2/100 vs 1/99; Return to operation room: 3/100 vs 3/99; Readmission: 0/100 vs 3/99
Tetro and Rudan, 2001 [34]	63	Tourniquet (n=33)	No tourniquet (n=30)	Superficial infection: 4/33 vs 1/30; Blistering: 1/33 vs 0/30; Haematoma: 6/33 vs 3/30; GI haemorrhage: 0/33 vs 1/30

VTE: Events were infrequent. Several trials reported isolated cases of DVT or PE without consistent differences between groups [6-8,20,23,24,27].

Wound complications: Tetro and Rudan found higher rates of superficial infection, haematoma, and blistering with tourniquet use [34]. Other studies, including Goel et al. [6], Dragosloveanu et al. [24], and Rantasalo et al. [20], found small numbers without clear differences. Tan et al. [8] and Liu et al. [32] both reported more wound problems in the tourniquet group (ooze, erythema, blisters, infection).

Local tourniquet-related effects: Tourniquet use was consistently associated with more pain and soft-tissue effects. Tai et al. [5], Goel et al. [6], Ran et al. [25], Yasin et al. [28], and Liu et al. [32] all reported higher thigh/knee pain or blistering. Stocks et al. described more MRI-confirmed quadriceps strain with tourniquet (22/49 vs 13/48; OR 2.7, 95%CI 1.1-6.7) [26]. Vandenbussche et al. found less early pain and better early knee flexion without tourniquet [30].

Systemic complications: These were rarely reported. Rantasalo et al. identified single cases of MI, arrhythmia, or PE without group differences [20]. Dennis et al. found a PE in no-tourniquet limbs and one wound dehiscence with tourniquet [7]. Tetro and Rudan noted one GI bleed in the no-tourniquet arm [34].

Functional outcomes: Dennis et al. [7] and Hung et al. [27] reported slower quadriceps recovery with tourniquet use, while Stocks et al. [26] found more quadriceps strain. Vandenbussche et al. found better early flexion without tourniquet, but no long-term differences [30].

Across these trials, the use of a tourniquet did not reduce thromboembolic events or systemic complications, but was associated with higher early thigh pain, quadriceps dysfunction, and a possible trend towards increased wound complications.

Tourniquet Throughout versus Limited/Timed Application RCTs

Four RCTs evaluated limited application strategies (Table 3), such as inflation only during cementation or shorter duration, compared with continuous inflation.

Blood transfusion and blood loss: The trial by Mittal et al. (n=65) was terminated early due to excess transfusion requirements in the cementation-only group (10/31 vs 2/34, OR 7.38, p=0.015) [9].

VTE: Diri et al. (n=62) found no significant difference in screened DVT (2/31 vs 1/31) [22]. Tarwala et al. (n=79) reported no DVT events [10].

Wound complications: Chaiyakit et al. (n=90) found higher wound complication rates in the continuous tourniquet arm (7/45 vs 0/45) [11]. Other studies reported small numbers of wound drainage or blistering, without consistent differences.

Serious complications: Tarwala et al. reported one case of compartment syndrome requiring fasciotomy in the tourniquet group [10].

Systemic complications: Mittal et al. recorded myocardial infarction (3/31), stroke (1/31), and GI bleed (1/31) in the short-duration arm, but event numbers were too small to infer differences [9].

Limited/timed use strategies reduced some local complications (eg, Chaiyakit et al. [11]) but may increase transfusion requirements (eg, Mittal et al. [9]). Evidence is inconsistent, with rare but important serious events reported.

**Table 3:** Randomised controlled trials – tourniquet throughout vs limited application (timing variations) DVT: deep vein thrombosis; MI: myocardial infarction; MUA: manipulation under anaesthesia; NR: not reported in the source publication; VTE: venous thromboembolism

Study (Author, Year)	Total participants	Arm A	Arm B	Key Findings / Complications Reported
Chaiyakit et al., 2024 [11]	90	Tourniquet throughout (n=45)	Cementation-only (n=45)	Wound complications: 7/45 vs 0/45; VTE: 0/45 vs 0/45
Diri et al., 2023 [22]	62	Tourniquet throughout (n=31)	Cementation-only (n=31)	DVT: 2/31 vs 1/31; Infection: 3/31 vs 2/31; Transfusion: 5/31 vs 9/31
Tarwala et al., 2014 [10]	79	Tourniquet throughout (n=39)	Cementation-only (n=40)	Compartment syndrome: 1/39 vs 0/40; DVT: 0/39 vs 0/40; MUA <90°: 1/39 vs 1/40; Wound drainage: 1/39 vs 0/40
Mittal et al., 2012 [9]	65	Long (incision → post-cement) (n=34)	Short (cementation only) (n=31)	Transfusion: 2/34 vs 10/31 (p=0.015); DVT: 1/34 vs 2/31; MI: 0/34 vs 3/31; Stroke: 0/34 vs 1/31; Death: 0/34 vs 1/31

Alternative Tourniquet Pressure Strategies and Three-Arm Trials

Three RCTs directly compared different pressure regimens or included three study arms (Tables 4, 5).

**Table 4:** Randomised controlled trials – tourniquet pressure variations DVT: deep vein thromobosis; PE: pulmonary embolism; LOP: limb occlusion pressure; SBP: systolic blood pressure

Study ID	N (total)	Arm A	Arm B	Key Findings / Complications Reported
Natesan 2024 [18]	311	LOP method (n = 154)	SBP +150 mm Hg method (n = 157)	Mean cuff pressures lower in LOP; less postoperative thigh pain with LOP; improved early range of motion; rare complications (incident rates low)
Olivecrona 2012 [12]	164	LOP-based pressure (n = 80)	Fixed high pressure (>225 mm Hg) (n = 84)	Discharge: 47 wound complications, 40/47 had cuff >225 mm Hg; at 2 months: 16 complications, 14/16 >225 mm Hg. Patients with cuff ≤225 mm Hg had no infections. No overall statistically significant difference between pressure groups in wound complication incidence
Kim 2019 [29]	160	Lower cuff pressure (SBP+120, n=80)	Conventional cuff pressure (SBP+150, n=80)	Skin complications: 2/80 vs 3/80; DVT: 1/80 vs 2/80; PE: 0/80 vs 0/80; Nerve palsy: 0/80 vs 0/80

**Table 5:** Randomised controlled trials – three-arm designs AOP: arterial occlusion pressure; DVT: deep vein thrombosis; IV: intravenous; PE: pulmonary embolism; SBP: systolic blood pressure; TQ: tourniquet; TXA: tranexamic acid; VAS: visual analogue scale.

Study (Author, Year)	Total Participants	Arm A	Arm B	Arm C	Key Findings/Complications Reported
Huang et al., 2017 [31]	150	A: TQ + IV + Topical TXA (n=50)	B: No TQ + IV + Topical TXA (n=50)	C: TQ only (n=50)	Intramuscular venous thrombosis: 6/50 vs 4/50 vs 3/50; Superficial infection: 1/50 vs 0/50 vs 3/50; Wound secretion: 6/50 vs 0/50 vs 9/50; Blistering: 0/50 vs 0/50 vs 3/50
Yi et al., 2021 [4]	150	Full tourniquet (n=50)	No tourniquet (n=50)	Cementation-only (n=50)	Calf muscle venous thrombosis: 2/50 vs 0/50 vs 1/50; Superficial cellulitis: 1/50 vs 0/50 vs 0/50; DVT/PE/Fracture: 0/50 vs 0/50 vs 0/50
Wu et al., 2022 [13]	138	A: AOP-based (n=46)	B: SBP+100 mmHg (n=45)	C: Fixed 300 mmHg (n=47)	Thigh pain (VAS Day 1): 4.4 vs 4.9 vs 6.5; Skin blister: 0/46 vs 0/45 vs 2/47; Wound complication: 0/46 vs 0/45 vs 0/47

Pressure strategies: LOP- or AOP-based pressures consistently produced lower cuff pressures, less thigh pain, and better early ROM compared with fixed SBP-based settings. Kim et al. found no meaningful differences in DVT or skin events between SBP+120 and SBP+150 [29]. Olivecrona et al. showed that nearly all wound complications occurred when cuff pressure exceeded 225 mmHg, though the overall incidence was not statistically different [12]. Natesan et al. confirmed the benefits of LOP with very low complication rates [14].

Three-arm trials: Events were generally infrequent. Yi et al. [4] reported occasional calf muscle thrombosis and minor superficial infections, while Huang et al. [31] found small numbers of intramuscular thromboses and wound issues without a clear advantage for the full tourniquet. Wu et al. demonstrated higher pain scores and blistering in the fixed 300 mmHg arm compared with lower-pressure strategies [13].

Individualised or lower-pressure regimens reduce cuff pressure-related pain and wound morbidity without increasing DVT or PE. Three-arm comparisons reinforce that omitting or limiting tourniquet use does not worsen thromboembolic outcomes.

Risk of Bias Assessment

**Figure 2:**
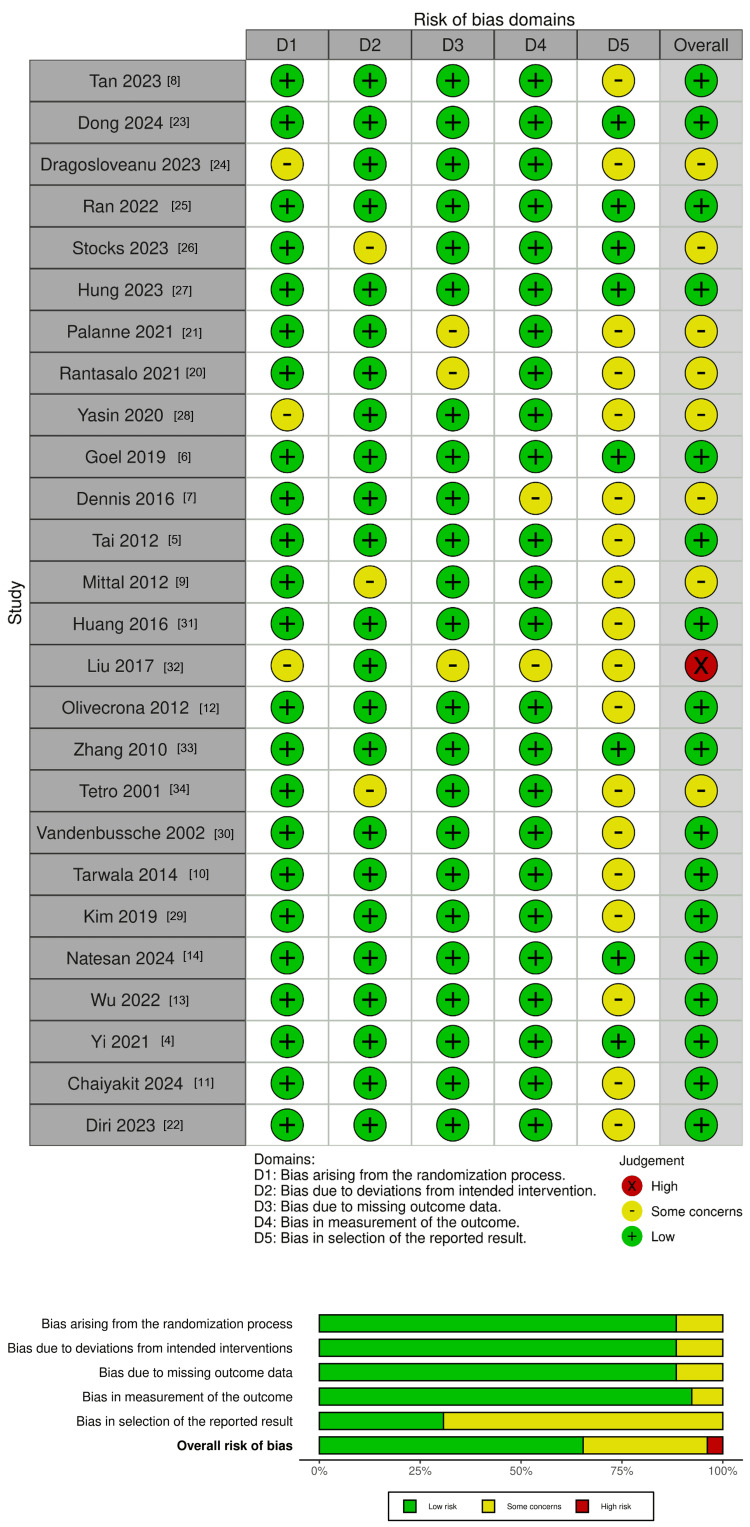
Risk of bias assessment of included studies. (A) Traffic-light plot showing the risk of bias for each of the 26 included RCTs across five Cochrane RoB 2 [18] domains: randomisation process (D1), deviations from intended interventions (D2), missing outcome data (D3), measurement of the outcome (D4), and selection of the reported result (D5). (B) Weighted bar plot displaying the proportion of studies rated at low risk, some concerns, or high risk of bias within each domain and overall. References: [4-14,20-34] Figure generated using robvis tool by McGuinness and Higgins, 2021 [35].

The 26 included RCTs were assessed using the Cochrane Risk of Bias 2 (RoB 2) tool. Most studies used adequate randomisation. Blinding was rarely feasible, raising detection bias for pain outcomes. Attrition was generally low. Definitions of complications varied. Overall, risk was judged low-to-moderate (Figure 2).

Outcomes

**Figure 3:**
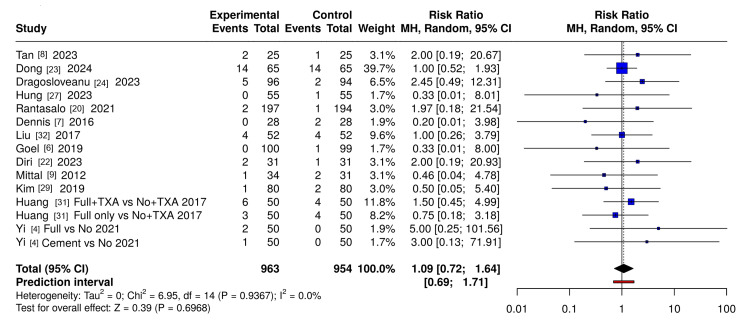
Forest plot of VTE in RCTs comparing tourniquet strategies in total knee arthroplasty Forest plot of RCTs assessing the effect of tourniquet use on VTE in total knee arthroplasty (TKA). Relative risks (RRs) were pooled with a random-effects model (REML) using inverse-variance weights, shown on a logarithmic scale with 95% confidence intervals (CIs). A 0.5 continuity correction was applied to single-zero studies, while double-zero studies were excluded. The dashed vertical line indicates no effect (RR = 1). Figure generated using trial data extracted by the authors and plotted with MetaAnalysisOnline (https://metaanalysisonline.com/) RCT: randomized controlled trials; VTE: venous thromboembolism

VTE: Meta-analysis of 15 RCT comparisons (n≈2000 patients) found no significant difference in VTE risk between tourniquet and alternative strategies (RR 1.09, 95%CI 0.72-1.64; I²=0%) (Figure 3).

Wound Complications

**Figure 4:**
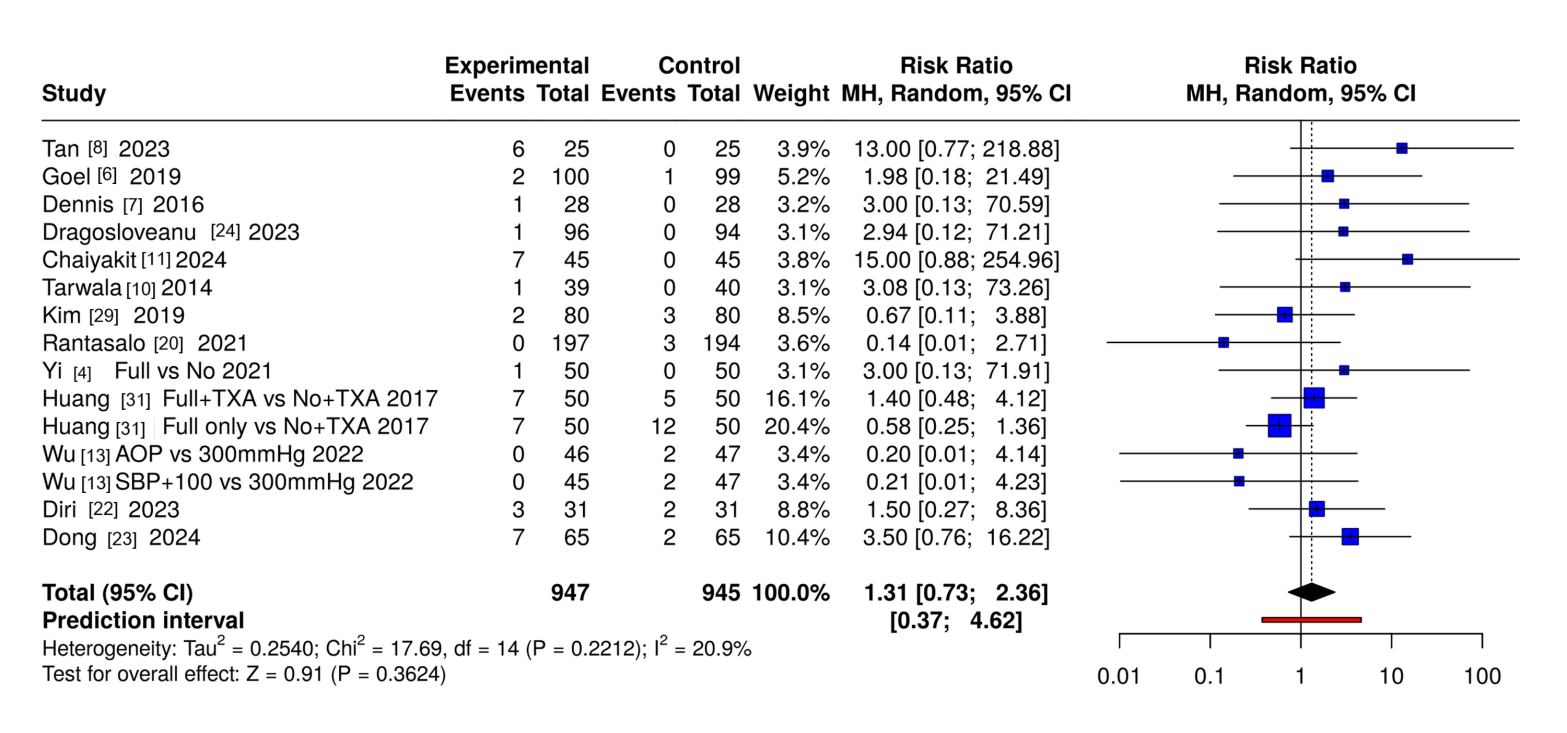
Forest plot of wound complications in randomised controlled trials comparing tourniquet strategies in total knee arthroplasty Forest plot of randomized controlled trials evaluating the effect of tourniquet use on wound complications following total knee arthroplasty. Relative risks (RRs) were pooled using a random-effects model (REML) with inverse-variance weighting and are displayed on a logarithmic scale with 95% confidence intervals (CIs). A continuity correction of 0.5 was applied to single-zero studies; double-zero studies were excluded from the pooled analysis. The dashed vertical line indicates no effect (RR = 1). Figure generated using extracted trial data with MetaAnalysisOnline (https://metaanalysisonline.com/)

Meta-analysis of 15 RCT comparisons (n≈2000 patients) found no statistically significant difference in wound complication risk between groups (RR 1.31, 95%CI 0.73-2.36; I²=20.9%) (Figure 4).

Blood Transfusion

**Figure 5:**
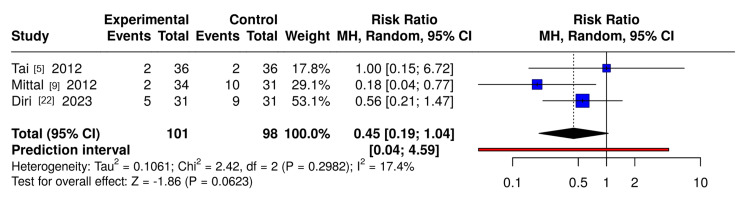
Forest plot of blood transfusion events in randomised controlled trials comparing tourniquet strategies in total knee arthroplasty Forest plot of randomized controlled trials (RCTs) investigating the impact of tourniquet use on the need for blood transfusion in total knee arthroplasty (TKA). Pooled relative risks (RRs) were estimated with a random-effects model (REML), weighted by the inverse variance. Results are presented on a logarithmic scale with 95% confidence intervals (CIs). A 0.5 continuity correction was applied to single-zero studies, and double-zero studies were not included in the pooled estimate. The dashed vertical line denotes the line of no effect (RR = 1). Figure created using extracted trial data with MetaAnalysisOnline (https://metaanalysisonline.com/)

Meta-analysis of three RCTs (n≈200 patients) (Figure 5) showed no significant difference overall (RR 0.45, 95%CI 0.19-1.04), with point estimates favouring limited/optimized tourniquet strategies due to the early-stopped Mittal et al.'s trial [9].

Certainty of evidence for each outcome was appraised using the GRADE approach (Table 6) [15].

**Table 6:** Summary of findings and certainty of evidence (GRADE) ⬤⬤⬤⬤ = High; ⬤⬤⬤◯ = Moderate; ⬤⬤◯◯ = Low; ⬤◯◯◯ = Very low RR: risk ratio; CI: confidence interval; RCT: randomized controlled trial; TQ: tourniquet Reference: GRADE Working Group methodology [19].

Outcome	No. of participants (studies)	Relative effect (95% CI)	Certainty of the evidence (GRADE)	Comments
Venous thromboembolism (VTE)	~2,000 (15 RCT comparison)	RR 1.09 (0.72–1.64)	⬤⬤◯◯ Low	Events were rare; CIs wide and compatible with benefit or harm. Downgraded for imprecision and detection bias (variable screening methods, not always systematic).
Wound complications (infection, blistering, delayed healing, wound drainage)	~2,000 (15 RCT comparison)	RR 1.31 (0.73–2.36)	⬤⬤⬤◯ Moderate	Direction toward more complications with tourniquet, but CI includes null. Downgraded for risk of bias (lack of blinding in outcome assessment) and inconsistency (moderate heterogeneity, I²=20.9%).
Blood transfusion requirement	~200 (3 RCTs with usable data)	RR 0.45 (0.19 to 1.04)	⬤⬤◯◯ Low	Point estimate suggests fewer transfusions with limited/optimised tourniquet, but effect driven by one small trial (Mittal 2012). Wide CI crossing null. Downgraded for imprecision and trial size.
Quadriceps dysfunction (pain/strain/strength deficits)	~700 (4 RCTs)	— (narrative synthesis only; no pooled estimate)	⬤⬤◯◯ Low	Consistent pattern of worse pain, delayed recovery, and MRI-confirmed strain with tourniquet, but outcome measures heterogeneous; downgraded for inconsistency and imprecision.
Pain (postoperative thigh/knee)	~1,100 (7-8 RCTs)	Not pooled (reported as VAS scores, higher with TQ)	⬤⬤◯◯ Low	Nearly all RCTs reported higher pain in TQ group in first 1–7 days, but scales and reporting varied; downgraded for inconsistency and risk of bias.
Functional recovery (ROM, straight-leg raise, rehab milestones)	~950 (6 RCTs)	Narrative — delayed early recovery with TQ	⬤⬤◯◯ Low	Consistent evidence of slower quadriceps activation and delayed milestones; long-term ROM equivalent; downgraded for heterogeneity in definitions and measurement.
Systemic complications (MI, arrhythmia, stroke, GI bleed, death)	~750 (5 RCTs, rare events)	Very sparse, no pooled estimate	⬤◯◯◯ Very Low	Events were extremely rare and scattered (single cases of MI, arrhythmia, GI bleed, death); evidence very uncertain.

Discussion

Venous Thromboembolism

Across 15 RCT datasets, thromboembolic events were rare and evenly distributed between groups. Trials evaluating full-duration tourniquet use [6-8,20,21,24,27,32], as well as selective, limited-duration, or pressure-optimised approaches [4,9,22,29,31], reported only isolated cases of deep vein thrombosis or pulmonary embolism, with no consistent differences between groups.

Wound Complications

Wound morbidity tended to be higher with tourniquet use, particularly with prolonged or high-pressure inflation, although pooled estimates were imprecise and not statistically significant. Early work found more infection and blistering in tourniquet groups [34]. More recent studies confirmed higher wound complication rates with continuous inflation compared with cement-only or no-tourniquet strategies [11,24]. Additional reports documented blistering or wound drainage [6,10]. Mechanistic studies reinforce this: Olivecrona et al. showed more wound problems at high fixed pressures (>225 mmHg) [12], Natesan et al. found less ecchymosis with limb-occlusion-based pressures [14], and Wu et al. observed blistering with 300 mmHg inflation [13]. Across 1,940 patients from 15 RCT comparisons, tourniquet use consistently increased wound morbidity, particularly with prolonged or high-pressure application [4,6-8,10,11,13,20,22-24,29,31].

Pain and Quadriceps Dysfunction

Six RCTs provided detailed assessments of muscle and pain outcomes: Dennis et al. reported persistent quadriceps weakness in tourniquet limbs [7], while Stocks et al. reported more MRI-confirmed quadriceps strain with tourniquet use (OR 2.7) [26]. Tai et al. demonstrated higher early thigh and knee pain scores [5], while Wu et al. showed a dose-response effect, with pain increasing at higher cuff pressures [13]. Hung et al. reported delayed quadriceps recovery [23], and Goel et al. found similar rates of thigh discomfort across groups [6]. Collectively, these trials indicate that tourniquet use consistently exacerbates early pain and impairs quadriceps function.

Rare and Systemic Complications

Serious but rare complications were also reported. Tarwala et al. described one compartment syndrome requiring fasciotomy [10], Dennis et al. noted wound dehiscence requiring reoperation [7], and Mittal et al. reported myocardial infarction, stroke, and gastrointestinal bleeding, though with very low event counts [9]. These underscore that tourniquet use is not entirely benign.

Pathophysiological Considerations

The adverse effects of tourniquet use arise from complex physiological mechanisms. Prolonged limb ischemia followed by reperfusion triggers oxidative stress, local inflammation, and microvascular damage, which contribute to soft-tissue oedema, delayed wound healing, and muscle necrosis [36,37]. These changes are intensified when cuff pressures exceed the minimum required for arterial occlusion, resulting in nerve compression and tissue hypoxia [29]. Histological and biochemical studies have confirmed elevated levels of reactive oxygen species and ischemia-reperfusion markers after tourniquet deflation, providing mechanistic support for the clinical findings of increased pain, swelling, and wound morbidity [36,37].

Comparison with Previous Literature

Our results are consistent with prior meta-analyses demonstrating higher rates of postoperative pain and wound complications associated with tourniquet use [15,16]. However, unlike earlier reviews that combined randomized and observational data, the present analysis focuses exclusively on RCTs and complication-specific outcomes. The absence of a clear reduction in transfusion requirements or thromboembolic risk reinforces that the routine use of a tourniquet provides limited benefit in the modern perioperative era, where tranexamic acid and meticulous haemostasis are standard [6,17].

Clinical Implications

The findings indicate that while tourniquet use can enhance intraoperative visualization, its routine full-duration application may not offer meaningful safety or functional advantages. Selective or time-limited use, such as during cementation, the adoption of limb occlusion pressure (LOP)-based protocols can maintain adequate exposure while minimizing ischemic tissue injury [14,29]. Such tailored strategies are consistent with enhanced recovery after surgery (ERAS) frameworks, which prioritize early mobilization, pain control, and preservation of muscle strength [38]. Consequently, tourniquet decisions should be individualized, balancing operative convenience against patient-centred outcomes.

Future Directions

Future trials should aim for multicentre collaboration and use standardized definitions of wound and muscle complications, stratified by tourniquet duration and pressure regimen. Integration of imaging and biomarker endpoints may clarify the causal relationship between ischemia-reperfusion injury and postoperative function [36,37]. In addition, cost-effectiveness and patient-reported outcome studies are needed to determine whether pressure-optimized or selective strategies translate into measurable improvements in recovery and healthcare utilization [38].

Limitations

This review has several limitations. Event rates for important outcomes such as VTE and systemic complications were very low, leading to wide confidence intervals and underpowered pooled estimates. Considerable heterogeneity in tourniquet strategies (full-duration, cement-only, variable pressures, contralateral-limb designs) complicates interpretation, even after subgroup analyses.

Definitions and methods of outcome assessment were inconsistent. VTE detection varied from routine screening to symptom-driven imaging, wound complications were defined variably, and quadriceps dysfunction was measured with different tools, limiting comparability.

The risk of bias across studies was generally moderate, as most were open-label, single-centre, and small in scale. Searches were restricted to three databases, the English language, and full-text availability, which may have missed unpublished, non-English, or non-indexed trials. Finally, generalisability is limited by differences in thromboprophylaxis, tranexamic acid use, and rehabilitation protocols across settings.

## Conclusions

Our review of randomized trials indicates that routine, full-duration tourniquet use during primary TKA offers limited safety advantages and is associated with trade-offs that include more early pain and muscle-related recovery issues. Approaches that limit duration or tailor cuff pressure appear more compatible with enhanced recovery pathways. In practice, tourniquet decisions should be individualized, balancing technical needs with patient-centred outcomes.

## Appendices

Ovid search strategy (2000-2025)

The following search strategy was used in Ovid (covering MEDLINE, Embase, and CENTRAL). The search was run in July 2025 and limited to human studies, English language, full-text availability, and publication years 2000-2025.

**Table 7:** Ovid search strategy (2000–2025)

Line	Query block	Ovid field scope
1	exp knee arthroplasty/	Subject heading
2	(total adj3 knee adj3 (arthroplast* or replac*)).ti,ab,kw.	Title/Abstract/Keyword
3	(TKA or TKR).ti,ab,kw.	Title/Abstract/Keyword
4	1 or 2 or 3	–
5	exp tourniquet/	Subject heading
6	tourniquet*.ti,ab,kw.	Title/Abstract/Keyword
7	((pneumatic or thigh) adj2 (tourniquet* or cuff)).ti,ab,kw.	Title/Abstract/Keyword
8	(tourniquet-free or tourniquetless or "no tourniquet").ti,ab,kw.	Title/Abstract/Keyword
9	5 or 6 or 7 or 8	–
10	exp postoperative complication/	Subject heading
11	(complication* or "adverse event*" or "adverse effect*" or AE or AEs or DVT or "deep vein thrombosis" or thromboembol* or "pulmonary embol*" or infection* or "wound complication*" or "wound problem*" or "skin necros*" or hematom* or ischem* or neuropath* or "nerve pals*" or "blood loss" or transfus* or "postoperative pain" or pain or swelling or edema or stiffness or "range of motion" or "implant fixation" or "cement penetration" or "radiolucent line*" or loos*).ti,ab,kw.	Title/Abstract/Keyword
12	10 or 11	–
13	randomized controlled trial/	Subject heading
14	random*.ti,ab.	Title/Abstract
15	(random* adj3 (trial or control* or allocat* or assign* or group* or arm*)).ti,ab.	Title/Abstract
16	13 or 14 or 15	–
17	4 and 9 and 12 and 16	–
18	meta analysis/	Subject heading
19	systematic review/	Subject heading
20	(systematic review or meta analy*).ti.	Title
21	18 or 19 or 20	–
22	17 not 21	–
23	limit 22 to english language	–
24	limit 23 to human	–
25	limit 24 to yr="2000 - 2025"	–
26	limit 25 to full text	–

The search retrieved 366 records. After title/abstract screening, 71 articles were assessed in full text, of which 26 randomized controlled trials (RCTs) met the inclusion criteria for the present review.
